# A Comparative Study of the Modified Baumann’s Angle Between the Dominant and Non-Dominant Sides in a North Indian Pediatric Population

**DOI:** 10.7759/cureus.47687

**Published:** 2023-10-25

**Authors:** Arpit Singh, Narendra S Kushwaha, Sanjiv Kumar, Rajnish Kumar, Ravindra Mohan, Shubhajeet Roy, Utkarsh Singh, Zohaib Abbas

**Affiliations:** 1 Orthopaedic Surgery, King George’s Medical University, Lucknow, IND; 2 Orthopaedic Surgery, King George's Medical University, Lucknow, IND; 3 Orthopaedic Surgery, Moti Lal Nehru Medical College, Prayagraj, IND; 4 Faculty of Medical Sciences, King George's Medical University, Lucknow, IND

**Keywords:** pediatric anatomy, pediatric orthopedics, forearm length, arm length, inter-epicondylar distance, radiological carrying angle, elbow carrying angle, modified baumann’s angle

## Abstract

Background

The literature points towards the fact that paediatric elbow fractures happen more frequently and have greater variability when contrasted with adults. Between 65%-75% of pediatric fractures involve the upper extremity, and supracondylar humerus is the most common of them all. To know the exact site of injury and to estimate the degree of reduction after manipulation, the modified Baumann’s angle, the Baumann’s angle, the Humero-condylar angle, the Anterior humeral line, and the Radio-capitellar line are the parameters most commonly used. This study was carried out to compare the modified Baumann’s angle between both upper limbs in the paediatric population.

Methodology

This cross-sectional study was conducted in a tertiary health care centre in Northern India for one year from September 1, 2021, to August 31, 2022. We included pediatric patients in the age group of 3-16 years. Age, sex, weight, height, BMI, secondary sexual characters, and handedness were noted in all the children enrolled in our study. In both the dominant and non-dominant sides, the mean arm length, the forearm length, the inter-epicondylar distance, the clinical carrying angle, the radiological carrying angle, and the modified Baumann’s angle were calculated.

Results

A total of 113 children were enrolled in the study. The majority of children (71.7%) had dominance on the right side. In both the dominant side and non-dominant side, mean arm length, forearm length, inter-epicondylar distance, clinical carrying angle, radiological carrying angle, and modified Baumann’s angle values were calculated. On evaluating the data statistically, a significant difference between the two sides was observed for all the parameters (p<0.05), except forearm length (p-value -0.954). Multivariate analysis showed that only BMI was significantly negatively associated with modified Baumann’s angle (p=0.016), and only age (0.019) and BMI (<0.001) were found to be significantly associated with the difference in modified Baumann’s angle.

Conclusions

The findings of this study will be helpful in the management of elbow disorders and their reconstruction following trauma. A significant difference was found in the modified Baumann’s angle between dominant and non-dominant sides, and it also showed a negative significant correlation with arm length, forearm length, and the presence of secondary sexual characteristics. The equations derived in this study will be helpful in the simple derivation of the modified Baumann’s angle and its difference from simple measurements of the upper limb parameters.

## Introduction

Elbow fractures have been seen to have a greater incidence and variability in the pediatric age group as compared to the adults [1]. Between 65%-75% of fractures in children affect the upper limb [2], and supracondylar fractures of the humerus have the lion’s share of 60% out of all upper limb fractures [3]. The percentage of elbow trauma shows a rising trend due to a surge in the levels of participation of this particular age group in recreational and competitive outdoor as well as indoor sports [4]. Even during the coronavirus disease 2019 (COVID-19) times, due to the lockdown, children found ways of recreational activities within the four walls of their homes, which further increased the incidence as well as the spectrum of elbow traumas. To assess the exact state of injury and to estimate the degree of reduction after manipulation, certain radiological criteria are employed, which include the modified Baumann’s angle and the Baumann’s angle in the Anterior-posterior view of the X-ray and the Humero-condylar angle, the Anterior humeral line and the Radio-capitellar line in the lateral view of the X-ray [5]. The Baumann’s angle was found to be a useful indicator of the adequacy of reduction of a displaced supracondylar humeral fracture while managing such patients by closed reduction and overhead traction of the olecranon [6]. Hence, it finds its application as an outcome measure for supracondylar fractures of Humerus in children [7]. However, there is limited information about the reliability of this measurement, with widely varying opinions in the existing literature. This study was conducted to compare the modified Baumann’s angle in the upper limbs of both sides in the pediatric population.

## Materials and methods

This cross-sectional study was conducted in a tertiary-level health care centre in Northern India for one year from September 1, 2021, to August 31, 2022. We included pediatric patients in the age group of 3-16 years who attended our hospital for some other non-traumatic complaints and children who/whose parents volunteered to participate in the study. We excluded children who had a history of orthopedic Trauma, history of orthopedic tumours, history of bone or joint Infections, history of surgery, and history of burns to either of the upper limbs. We also excluded patients who had post-polio residual paralysis, patients with metabolic bone diseases like rickets, Osteogenesis Imperfecta, Fibrous Dysplasia, patients suffering from neurological conditions with abnormal muscle tone due to cerebral palsy, head injury, or motor neuron disease, patients of arthritis like rheumatoid arthritis, juvenile rheumatoid arthritis, congenital orthopedic deformities, or children who had not developed hand preference at the time of enrollment in the study. 

**Figure 1:**
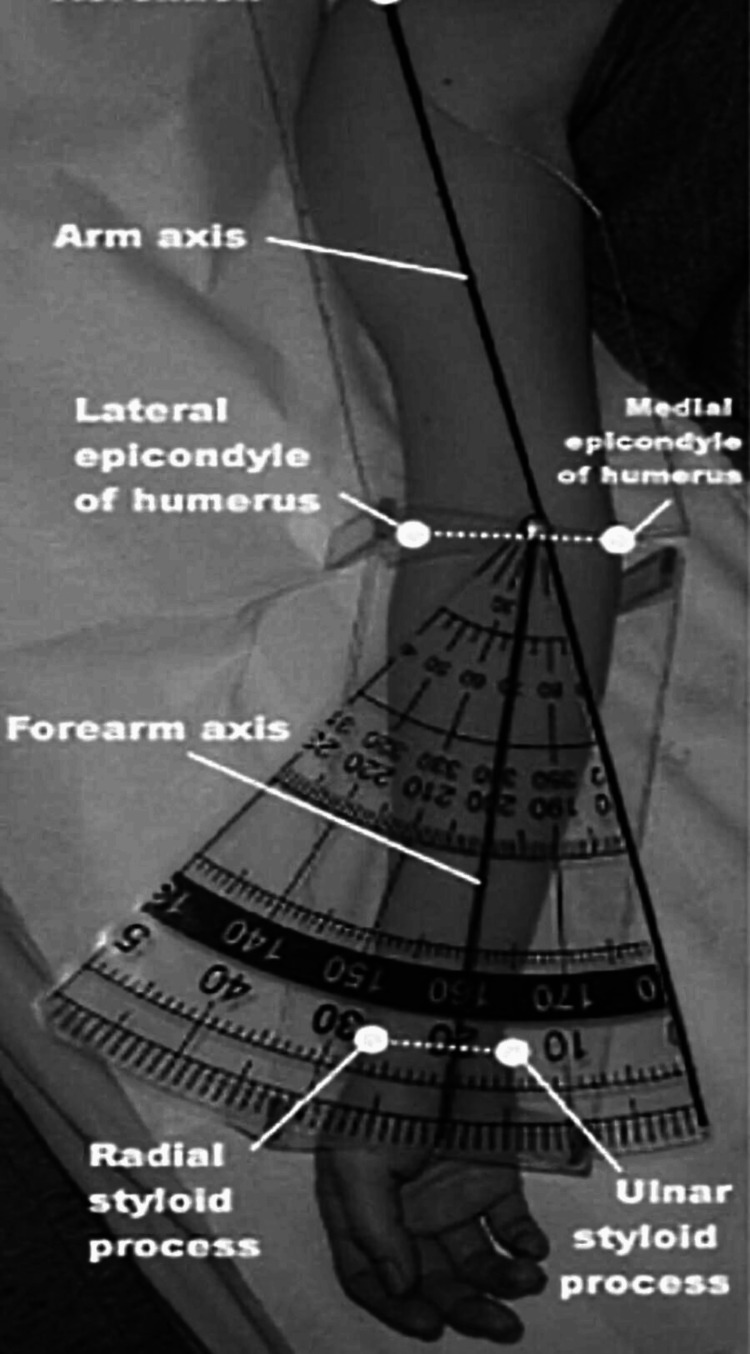
Measurement of the clinical carrying angle

Age, sex, weight, height, BMI, secondary sexual characters, and handedness were noted in all the children enrolled in our study. In both the dominant side and non-dominant sides, the mean arm length, the forearm length, the inter-epicondylar distance, the clinical carrying angle, the radiological carrying angle, and the modified Baumann’s angle (on anteroposterior (AP) view of X-ray of the elbow joint) were calculated. The clinical carrying angle was measured with full extension of the elbow and full supination of the forearm. The angle between the central axis of the forearm and arm was measured using manual goniometry. The central axis of the forearm was taken as a line joining the midpoint of the inter-epicondylar line to the midpoint of the inter-styloid line at the wrist and the central axis of the arm was taken as a line joining the midpoint of the inter-epicondylar line to the tip of the acromion process of the arm (Figure 1).

**Figure 2:**
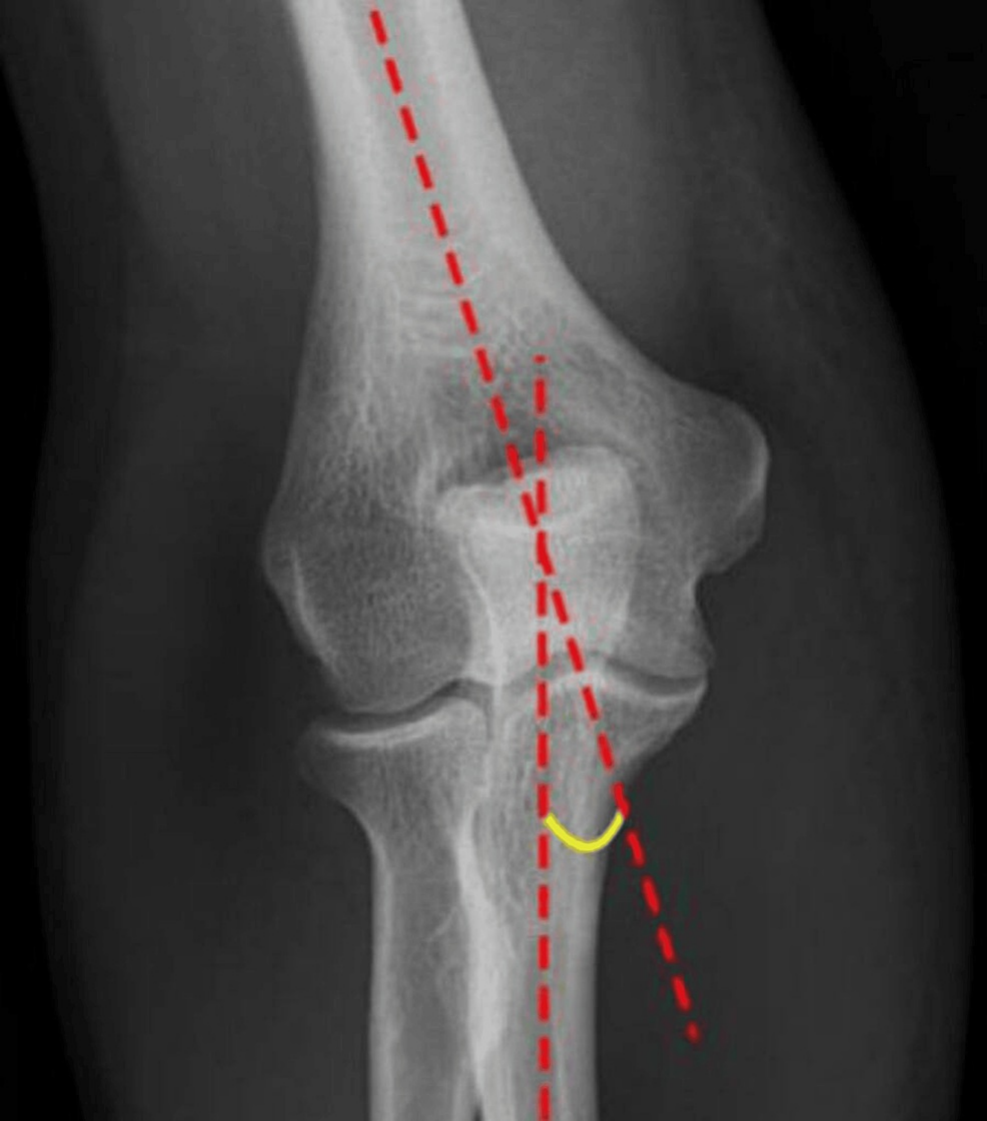
Measurement of the radiological carrying angle The radiological carrying angle is shown in yellow.

The radiological carrying angle was measured on a true AP view of the X-ray of the elbow as the angle between the longitudinal axis of the shaft of the humerus and a longitudinal line drawn along the shaft of the ulna. The axes of the humerus and ulna were determined by two central points on the shafts of the respective bones (Figure 2).

**Figure 3:**
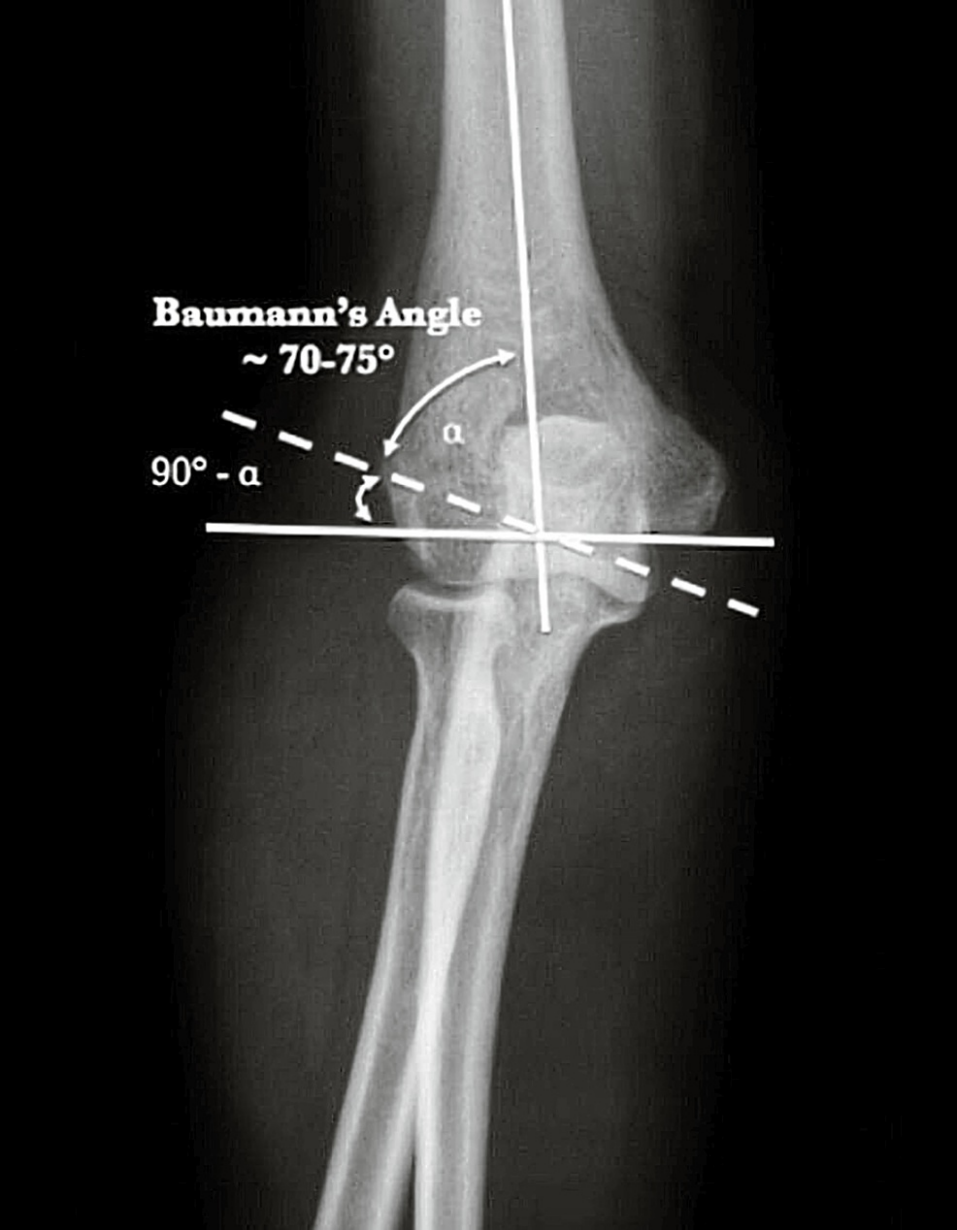
Measurement of modified Baumann's angle

Modified Baumann’s angle was measured on a true AP view X-ray of the elbow. A straight line was drawn through the middle of the humeral shaft by taking two central points on the humeral shaft and a second line was drawn perpendicular to the humeral shaft. A third line was drawn along the lateral condyle growth plate. The angle between the 1st line and the 3rd line gave us the Baumann’s angle. The angle between the 2nd and 3rd lines gave us modified Baumann’s angle (Figure 3).

To measure the inter epicondylar distance, the arm was first lifted to the level of the shoulder and the forearm was flexed to 90 degrees to make the humeral epicondyles prominent and easily palpable. A Vernier calliper was used to measure the inter-epicondylar distance. The fixed arm of the calliper was placed on the lateral epicondyle and the movable arm was then adjusted to the medial epicondyle. Forearm length was measured as the distance from the mid-point of a line joining the radial and ulnar styloid to the mid-point of a line joining the medial and lateral epicondyles of the humerus using a standard measuring tape, with the arm flexed to 90 degrees. The arm length was measured as the distance from the tip of the acromion process to the mid-point of a line joining the medial and lateral epicondyles of the humerus using a standard measuring tape. The height was calculated in a standing, erect, anatomical position barefoot from the vertex to the heel using a measuring tape.

The sample size was calculated as 110, considering Baumann’s angle in the common range of 71 - 800, with a prevalence of 60%, as observed in the study by Awasthi et al. (2017) [8]. Data was analysed using SPSS software, version 24.0 (IBM Corp., Armonk, NY). The ANOVA test, the paired t-test, and the independent samples t-test were used to analyse the data. Pearson correlation coefficient was used to evaluate bivariate linear correlation. Linear regression was performed for multivariate analysis. A p-value less than 0.05 was considered statistically significant.

Ethical clearance was taken from the Institutional Ethics Committee of King George’s Medical University (Ref No.: 103rd ECM IIB-Thesis/P34, dated 2020 Dec 10) before beginning the study, and written informed consent was taken from the parents of the children and permission was also taken if the age of the child was above or equal to 18 years, agreeing to participate in this study.

## Results

**Figure 4:**
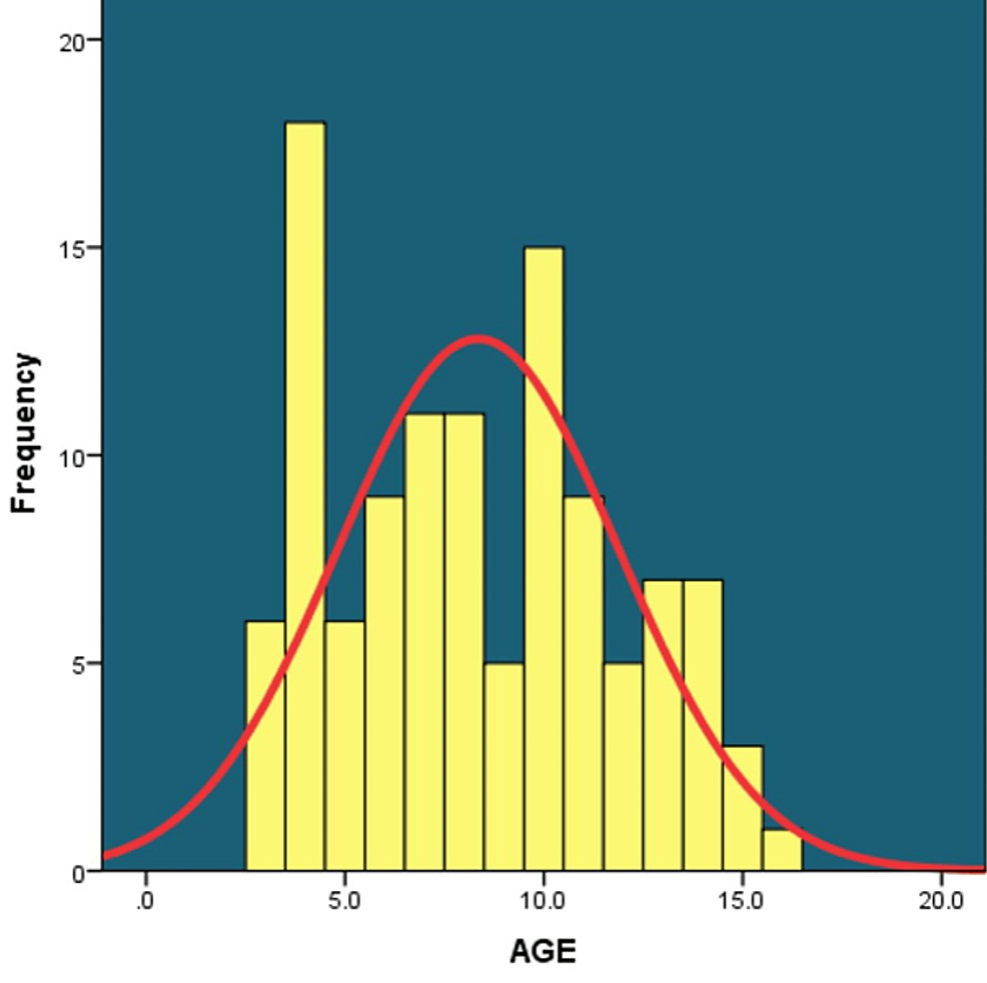
Age profile of the study population

A total of 113 children were enrolled in the study in the age group of 3 to 16 years. The majority of them were boys (n=78, 69.03%), while the remaining (n=35, 30.97%) were girls. The mean age of the children was 8.35±3.52 years. Maximum (n=34; 30.1%) children were in the age group of 9-12 years, followed by those in the 6-8 years age group (n-31; 27.4%), 3-5 years age group (n=30; 26.5%) and >12 years age group (n-18; 15.9%). Among boys too, the maximum (n=25; 32.1%) were aged 9-12 years followed by those aged 6-8 years (n=23; 29.5%), 3-5 years (n=17; 21.8%) and >12 years (n=13; 16.7%) respectively. The mean age of boys was 8.62±3.34 years. However, among girls, maximum (n=13; 37.1%) were aged 3-5 years followed by those aged 9-12 years (n=9; 25.7%), 6-8 years (n=8; 22.9%) and >12 years (n=5; 14.3%) respectively. The mean age of girls was 7.74±3.88 years. Although the mean age of boys was higher as compared to that of girls, this difference was not significant statistically (p=0.225) (Table 1) (Figure 4).

**Table 1:** Age and sex profile of study population (n=113) p<0.05 was considered significant

Age Group	Total (N=113)		Males (n=78 (69.03%))		Females (n=35 (30.97%))	
	No.	%	No.	%	No.	%
3-5 Years	30	26.5	17	21.8	13	37.1
6-8 Years	31	27.4	23	29.5	8	22.9
9-12 Years	34	30.1	25	32.1	9	25.7
>12 Years	18	15.9	13	16.7	5	14.3
Mean age±SD (Range) in years	8.35±3.52 (3-16)		8.62±3.34 (3-16)		7.74±3.88 (3-15)	
	t=1.220; p=0.225					

The mean body weight was 24.0±7.9 kg (range: 12-43 kg). The heights of children ranged from 78-156 cm with a mean of 120.6±19.9 cm. The BMIs of children ranged from 11.6 to 21.7 kg/m^2^ with a mean BMI of 16.1±2 kg/m^2^. The majority of the children (n=81, 71.7%) had hand dominance on the right side. The presence of secondary sexual characteristics was seen in 51 (45.1%) children (Table 2). 

**Table 2:** Anthropometric details, dominant side, and presence of secondary sexual characteristics of the study population

Variable	Mean	SD	Range	
			Minimum	Maximum
Weight (kg)	24.0	7.9	12	43
Height (cm)	120.6	19.9	78.0	156.0
BMI (kg/m^2^)	16.1	2	11.6	21.7
	N		%	
Dominant side				
Left	32		28.3	
Right	81		71.7	
Secondary sexual characteristics				
Absent	62		54.9	
Present	51		45.1	

On the dominant side, mean arm length, forearm length, inter-epicondylar distance, clinical carrying angle, radiological carrying angle, and modified Baumann’s angle values were 21.79±3.06 cm, 18.88±6.72 cm, 6.72±0.81 cm, 9.54±1.66°, 9.57±1.67° and 17.98±4.38° respectively. On the other hand, on the non-dominant side, mean arm length, forearm length, inter-epicondylar distance, clinical carrying angle, radiological carrying angle, and modified Baumann’s angle values were 21.78±3.05 cm, 18.88±3.06 cm, 6.71±0.82 cm, 9.82±1.70°, 9.82±1.69° and 17.78±4.16° respectively. On evaluating the data statistically, a significant difference between the two sides was observed for all the parameters (p<0.05) except forearm length (p=0.954). Among boys, a significant difference between the two sides was observed for all the parameters (p<0.05) except for arm length (p=0.063) and forearm length (p=0.954). Among girls, a significant difference between the two sides was observed for the clinical carrying angle and radiological carrying angle (p<0.001) respectively. For none of the other parameters the difference between the two groups was significant (p>0.05) (Table 3).

**Table 3:** Comparison of linear measurements and modified Baumann’s angle between dominant and non-dominant sides

	Overall								Males								Females							
Parameter	Dominant Side			Non-Dominant side			Significance of difference		Dominant Side			Non-Dominant side			Significance of difference		Dominant Side			Non-Dominant side			Significance of difference	
	Mean± SD	Range		Mean± SD	Range		‘t’	‘p’	Mean± SD	Range		Mean± SD	Range		‘t’	‘p’	Mean± SD	Range		Mean± SD	Range		‘t’	‘p’
		Minimum	Maximum		Minimum	Maximum				Minimum	Maximum		Minimum	Maximum				Minimum	Maximum		Minimum	Maximum		
Arm length (cm)	21.79±3.06	15.7	31.0	21.78±3.05	15.7	30.5	2.133	0.035*	229±3.07	16.2	31.0	227±3.06	16.2	30.5	1.887	0.063	21.13±2.97	15.7	26.4	21.13±2.98	15.7	26.4	1.435	0.160
Forearm length (cm)	18.88±3.07	13.0	27.0	18.88±3.06	13.40	27.00	-0.057	0.954	19.05±3.06	13.0	27.0	19.05±3.04	14.0	27.0	-0.004	0.997	18.50±3.12	13.4	24.8	18.51±3.11	13.4	24.8	-1.474	0.150
Inter-epicondylar distance (cm)	6.72±0.81	4.20	8.10	6.71±0.82	4.10	8.10	2.495	0.014*	6.79±0.80	4.2	8.1	6.78±0.81	4.1	8.1	2.131	0.036*	6.56±0.81	4.7	7.9	6.56±0.82	4.7	7.9	1.435	0.160
Clinical carrying angle (^o^)	9.54±1.66	5.4	13.4	9.82±1.70	6.0	13.6	-8.691	<0.001*	9.57±1.58	5.4	12.6	9.84±1.63	6.0	12.8	-6.338	<0.001*	9.46±1.85	6.5	13.4	9.79±1.88	7.1	13.6	-6.613	<0.001*
Radiological carrying angle (^o^)	9.57±1.67	5.50	13.40	9.82±1.69	6.1	13.6	-7.961	<0.001*	9.60±1.58	5.5	12.7	9.83±1.60	6.1	12.8	-5.971	<0.001*	9.51±1.87	6.6	13.4	9.81±1.90	7.1	13.6	-5.470	<0.001*
Modified Baumann’s angle (^o^)	17.98±4.38	9.50	28.20	17.78±4.16	9.60	28.20	2.184	0.031*	18.39±4.33	10.4	28.2	18.10±4.18	10.6	28.2	2.510	0.014*	17.07±4.41	9.5	25.4	17.05±4.06	9.6	22.6	0.119	0.906

For all the parameters mean values were higher in males as compared to that in females; however, this difference was significant statistically only for arm length. On the dominant side, for all the parameters mean values were higher in males as compared to that in females; however, this difference was not statistically significant for any of these parameters. On the non-dominant side, for all the parameters mean values were higher in males as compared to that in females; however, this difference was significant statistically for any of these parameters (Table 4).

**Table 4:** Comparison of linear measurements and modified Baumann’s angle between males and females

	Both sides combined						Dominant Side						Non-Dominant Side					
Parameter	Males (n=156)		Females (n=70)		Significance of difference (Independent samples ‘t’-test)		Males (n=156)		Females (n=70)		Significance of difference (Independent samples ‘t’-test)		Males (n=156)		Females (n=70)		Significance of difference (Independent samples ‘t’-test)	
	Mean	SD	Mean	SD	‘t’	‘p’	Mean	SD	Mean	SD	‘t’	‘p’	Mean	SD	Mean	SD	‘t’	‘p’
Arm length (cm)	228	3.06	21.13	2.95	2.193	0.029*	229	3.07	21.13	2.97	1.550	0.124	227	3.06	21.13	2.98	1.538	0.127
Forearm length (cm)	19.05	3.04	18.51	3.07	1.218	0.225	19.05	3.06	18.50	3.12	0.868	0.388	19.05	3.04	18.51	3.11	0.847	0.399
Inter-epicondylar distance (cm)	6.78	0.81	6.56	0.81	1.938	0.054	6.79	0.80	6.56	0.81	1.378	0.171	6.78	0.81	6.56	0.82	1.350	0.180
Clinical carrying angle (^o^)	9.70	1.60	9.62	1.86	0.329	0.743	9.57	1.58	9.46	1.85	0.322	0.748	9.84	1.63	9.79	1.88	0.144	0.886
Radiological carrying angle (^o^)	9.71	1.59	9.66	1.88	0.227	0.820	9.60	1.58	9.51	1.87	0.254	0.800	9.83	1.60	9.81	1.90	0.068	0.946
Modified Baumann’s angle (^o^)	18.25	4.25	17.06	4.20	1.945	0.053	18.39	4.33	17.07	4.41	1.490	0.139	18.10	4.18	17.05	4.06	1.244	0.216

Among those aged 3-5 years, statistically significant differences were observed for arm length (p=0.043), inter-epicondylar distance (p=0.024), clinical carrying angle (p<0.001), and radiological carrying angle (p=0.001) respectively. Among those aged 6-8 years, statistically significant differences were observed for clinical and radiological carrying angles only, which were found to be significantly higher in non-dominant as compared to the dominant side. Among those aged 9-12 years, on the dominant side, statistically significant differences were observed for clinical and radiological carrying angles only, which were found to be significantly higher in non-dominant as compared to the dominant side. Among those aged >12 years, mean clinical and radiological carrying angles were found to be significantly higher in the non-dominant as compared to the dominant side while mean modified Baumann’s angle was found to be significantly higher in the dominant as compared to the non-dominant side (Table 5).

**Table 5:** Age-wise comparison of linear measurements and modified Baumann’s angle between dominant and non-dominant sides

	3-5 Years								6-8 Years								9-12 Years								>12 Years							
Parameter	Dominant Side			Non-Dominant side			Significance of difference (Paired ‘t’-test		Dominant Side			Non-Dominant side			Significance of difference (Paired ‘t’-test		Dominant Side			Non-Dominant side			Significance of difference (Paired ‘t’-test		Dominant Side			Non-Dominant side			Significance of difference (Paired ‘t’-test	
	Mean± SD	Range		Mean± SD	Range		‘t’	‘p’	Mean± SD	Range		Mean± SD	Range		‘t’	‘p’	Mean± SD	Range		Mean± SD	Range		‘t’	‘p’	Mean± SD	Range		Mean± SD	Range		‘t’	‘p’
		Minimum	Maximum		Minimum	Maximum				Minimum	Maximum		Minimum	Maximum				Minimum	Maximum		Minimum	Maximum				Minimum	Maximum		Minimum	Maximum		
Arm length (cm)	18.89±1.92	15.7	23.8	18.88±1.92	15.7	23.8	2.112	0.043*	20.85±1.68	16.2	24.1	20.84±1.67	16.2	24.1	1.438	0.161	22.86±1.92	19.6	28.9	22.85±1.92	19.6	28.9	0.892	0.379	26.24±1.91	23.8	31.0	26.21±1.83	23.8	30.5	1.000	0.331
Forearm length (cm)	15.91±1.60	13.0	19.4	15.98±1.48	13.4	19.4	-1.508	0.142	17.84±1.49	14.4	19.9	17.73±1.50	14.4	19.9	1.286	0.209	19.79±1.85	16.9	25.2	19.84±1.88	16.9	25.2	-.635	0.530	23.85±1.26	21.1	27.0	23.85±1.26	21.1	27.0	0.000	1.000
Inter-epicondylar distance (cm)	5.67±0.64	4.2	6.6	5.66±0.66	4.1	6.6	2.381	0.024*	6.80±0.48	6.0	7.9	6.80±0.48	6.0	7.9	1.438	0.161	7.15±0.29	6.0	7.7	7.15±0.29	6.0	7.7	0.000	1.000	7.49±0.30	7.0	8.1	7.48±0.31	7.0	8.1	1.000	0.331
Clinical carrying angle (^o^)	7.73±0.81	5.4	8.8	8.01±0.74	6.0	9.1	-4.058	<0.001*	8.86±0.73	7.4	10.4	9.08±0.78	7.9	11.0	-4.182	<0.001*	10.66±0.92	7.8	12	10.97±0.94	8.2	12.6	-5.965	<0.001*	11.58±0.92	9.0	13.4	11.93±1.12	8.2	13.6	-3.270	0.005*
Radiological carrying angle (^o^)	7.78±0.79	5.5	8.8	8.04±0.72	6.1	9.1	-3.568	0.001*	8.86±0.71	7.4	10.2	9.10±0.81	7.8	11.1	-4.058	<0.001*	10.70±0.96	7.8	12.4	10.98±0.96	8.2	12.6	-6.356	<0.001*	11.64±0.93	9.0	13.4	11.88±1.12	8.2	13.6	-2.372	0.030*
Modified Baumann’s angle (^o^)	18.78±4.57	9.5	25.4	18.49±4.22	9.6	23.3	1.108	0.277	18.20±4.08	11.2	25.4	18.11±3.84	11.5	24.7	0.726	0.474	17.62±4.54	10.4	28.2	17.60±4.49	10.6	28.2	0.258	0.798	16.97±4.34	11.5	24.6	16.37±3.87	10.9	24.3	2.219	0.040*

Though with increasing age there was a decreasing trend of modified Baumann’s angle values, but it was not significant statistically (p=0.150). Boys had higher mean modified Baumann’s angle value (18.25±4.25°) as compared to girls (17.06±4.20°) however this difference was not found to be significant statistically (p=0.053). Left-handed children (17.45±4.48°) had lower mean values as compared to right-handed children (18.05±4.17°) yet this difference was not found to be significant statistically (p=0.344). Those having secondary sex characteristics had lower mean values (17.02±4.38°) as compared to those not having secondary sex characteristics (18.58±4.04°) and this difference was also significant statistically (p=0.006).

The difference in modified Baumann’s angle between dominant and non-dominant sides did not show a significant association with age, sex, handedness, and secondary sex characteristics (p>0.05) (Table 6).

**Table 6:** Factors affecting modified Baumann’s angle and modified Baumann’s angle difference between dominant and non-dominant sides

	Modified Baumann’s Angle			Modified Baumann’s Angle Difference		
Factor	Mean Difference	±SD	Statistical significance	Mean Difference	±SD	Statistical significance
Overall mean difference±SD (^o^)	17.88	4.26		0.21	1.00	
Range (^o^)	9.50-28.20			-1.79-3.80		
Age						
3-5 Years (n=30)	18.63	4.36	f=1.791; p=0.150	0.30	1.46	f=1.572; p=0.200
6-8 Years (n=31)	18.15	3.93		0.09	0.67	
9-12 Years (n=34)	17.61	4.48		0.02	0.54	
>12 Years (n=18)	16.67	4.06		0.60	1.15	
Sex						
Male (n=78)	18.25	4.25	t=1.945; p=0.053	0.29	1.02	t=1.332; p=0.186
Female (n=35)	17.06	4.20		0.02	0.95	
Handedness						
Left (n=32)	17.45	4.48	t=0.949; p=0.344	0.14	1.02	t=0.454; p=0.651
Right (n=81)	18.05	4.17		0.23	1.00	
Secondary sexual characteristics						
Present (n=51)	17.02	4.38	t=2.793; p=0.006*	0.23	0.85	t=0.223; p=0.824
Absent (n=62)	18.58	4.04		0.19	1.12	

A weak negative yet significant correlation of modified Baumann’s angle was observed with arm length, forearm length, and clinical and radiological carrying angles. There was a weak negative correlation of modified Baumann’s angle with inter-epicondylar distance too but this was not significant statistically. Among other bivariate correlations, there was a weak positive correlation of arm length with forearm length, inter-epicondylar distance and clinical carrying angle, and a moderate and significant and positive correlation of forearm length with inter-epicondylar distance, clinical and radiological carrying angles. There is a strong positive correlation between inter-epicondylar distance with clinical and radiological carrying angles and a strong near-perfect correlation between radiological and clinical carrying angles.

**Figure 5:**
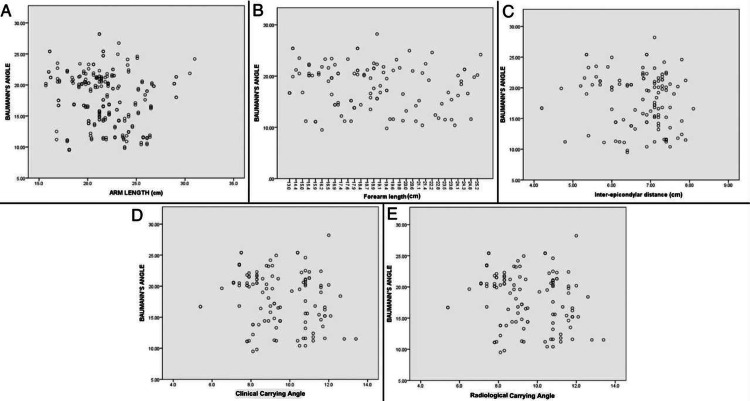
X-Y scatter plots X-Y scatter plots showing the correlation between modified Baumann's Angle with different linear and angular measurements: (A) arm length (cm); (B) forearm length (cm); (C) inter-condylar distance (cm); (D) clinical carrying angle (°); and (E) radiological carrying angle (°).

There was no significant correlation of difference in modified Baumann’s angle between dominant and non-dominant sides with arm length, forearm length, inter-epicondylar distance, clinical carrying angle, and radiological carrying angle (p>0.05) (Table 7) (Figure 5).

**Table 7:** Correlation of modified Baumann’s angle and modified Baumann’s angle difference between dominant and non-dominant sides Correlation of modified Baumann’s angle and modified Baumann’s angle difference between dominant and non-dominant sides with different linear and angular measurements (n=226) (Pearson correlation coefficient).

	Modified Baumann’s angle	Arm Length	Forearm length	Inter-epi-condylar distance	Clinical Carrying Angle	Radio-logical carrying angle		Modified Baumann’s angle Difference	Arm Length	Forearm length	Inter-epi-condylar distance	Clinical Carrying Angle	Radio-logical carrying angle
Modified Baumann’s angle	1	-0.140*	-0.166*	-0.127	-0.198*	-0.200*	Modified Baumann’s angle Difference	1	0.108	0.088	0.100	-0.005	-0.012
Arm Length		1	0.214*	0.202*	0.131*	0.130	Arm Length		1	0.214*	0.202*	0.131*	0.130
Forearm length			1	0.679*	0.752*	0.748*	Forearm length			1	0.679*	0.752*	0.748*
Inter-epi-condylar distance				1	0.705*	0.748*	Inter-epi-condylar distance				1	0.705*	0.748*
Clinical Carrying Angle					1	0.998*	Clinical Carrying Angle					1	0.998*
Radio-logical carrying angle						1	Radio-logical carrying angle						1

In a multivariate linear regression model where modified Baumann’s angle was projected as a dependent variable on independent variables age, sex, BMI, handedness, secondary sexual characteristics, arm length, forearm length, inter-epicondylar distance and clinical and radiological carrying angles only BMI emerged as the significant variable negatively associated with modified Baumann’s angle (p=0.016). The model had a low explanatory power (r2=0.096) thus implying that based on this multivariate model changes in modified Baumann’s angle could be explained by 9.6% only.

In a linear regression model, where the difference in modified Baumann’s angle between the dominant and non-dominant side was considered as a dependent variable on independent variables age, sex, handedness, secondary sex characteristics, arm length, forearm length, inter-epicondylar distance, clinical carrying angle and radiological carrying angle, only age, and BMI were found to be significantly associated with the dependent variable. The projected model had a limited explanatory ability (r2=0.132) (Table 8).

**Table 8:** Multivariate analysis of the factors affecting modified Baumann’s angle and modified Baumann’s angle difference

	Modified Baumann’s Angle			Difference in Modified Baumann’s Angle		
Factor	Beta Constant	SE	‘p’	Beta Constant	SE	‘p’
Alpha Constant	29.09	5.83	<0.001	3.286	1.342	0.015*
Age	0.290	0.255	0.256	0.139	0.059	0.019*
Sex (1=Male, 2=Female)	-1.033	0.620	0.097	-0.143	0.143	0.317
BMI	-0.348	0.143	0.016	-0.142	0.033	<0.001*
Handedness (1=Left, 2=Right)	0.632	0.625	0.313	0.051	0.144	0.722
Secondary sexual characteristics (1=Present, 2=Absent)	1.224	1.063	0.251	0.204	0.245	0.405
Arm Length	-0.018	0.023	0.437	0.001	0.005	0.877
Forearm length	-0.161	0.187	0.391	-0.010	0.043	0.822
Inter-epicondylar distance	-0.150	0.561	0.790	0.055	0.129	0.672
Clinical carrying angle	1.107	2.672	0.679	0.806	0.616	0.192
Radiological carrying angle	-1.639	2.668	0.540	-1.049	0.616	0.089
R^2^	0.096			0.132		

The derived equation for the projection of modified Baumann’s angle based on this model is-

Modified Baumann’s angle = 29.09 + (0.290xAge) - (1.033xSex) - (0.348xBMI) + (0.632xHandedness) + (1.224xSecondary sexual characteristics) - (0.018xArm Length) - (0.161xForearm length) - (0.150xInter-epicondylar distance) + (1.107xClinical carrying angle) - (1.639xRadiological carrying angle)

An equation was derived for the difference in Modified Baumann’s Angle between dominant and non-dominant hands-

Modified Baumann’s Angle Difference = 3.286 + (0.139xAge) - (0.143xSex) - (0.142xBMI) + (0.051xHandedness) + (0.204xSecondary sexual characteristics) + (0.001xArm Length) - (0.010xForearm length) + (0.055xInter-epicondylar distance) + (0.806xClinical carrying angle) - (1.049xRadiological carrying angle)

(Where: Age is in years; Sex: Male=1, Female=2; BMI is in kg/m^2^; Handedness: Left=1, Right=2; Secondary sexual characteristics: Present=1, Absent=2; Arm length is in cm; forearm length is in cm; inter-epicondylar distance is in cm; clinical carrying angle is in degrees; and radiological carrying angle is in degrees).

## Discussion

This cross-sectional study examined a cohort of 113 children aged between 3 and 16 years. The average age of the cases included in our study was 8.35±3.52 years. In a study conducted by Williamson et al. [9], a cohort of 114 children between the ages of 2 and 13 were included to investigate Baumann's angle. Similarly, Madison et al. [10] examined a group of 45 children, with 28.9% of them being under the age of 6, in their study. The study conducted by Dai L involved the enrollment of 98 children of Chinese descent, ranging in age from 2 to 13 years [11]. Keenan and Clegg [12] conducted a study examining the relationship between Baumann's angle and age, sex, and side and its potential implications for the radiographic surveillance of supracondylar fractures of the humerus. The study included an enrollment of 577 children aged 2 to 14 years. In their study on the anthropometric characterization of elbow angles and lines, Awasthi et al. [8] examined a cohort of 125 children from India, ranging in age from 3 to 13 years.

In the current study, the investigators found that the average modified Baumann's angle on the dominant side was 17.98±4.38°. In contrast, the average value on the non-dominant side was shown to be 17.78 ± 4.38°. In their research, Keenan and Clegg [12] determined the average Baumann's angle to be 73.5°± 5.78 on the left side and 73.7°± 7.47 on the right side. 

In our study, a significant difference was seen in the mean modified Baumann's angle between the dominant side (18.39±4.33°) and the non-dominant side (18.10±4.1°) in boys. In a study conducted by Keenan and Clegg [12], with a sample size of 577 youngsters, it was observed that there was no statistically significant difference in the average Baumann's angle when comparing the left and right sides. The observed difference could potentially be attributed to a variation in the racial composition of the individuals included in the study. The community examined by Keenan and Clegg [12] consisted of individuals of European origin, whereas our study focused on a population from northern India.

No significant difference was observed in the values of modified Baumann's angle with increasing age, which aligns with the findings of Keenan and Clegg [12]. Their study also reported no growing or decreasing trend in the values of Baumann's angle throughout the years, regardless of sex or side. Dai et al. [11] conducted another study that similarly concluded that there were no statistically significant variations in Baumann's angles with advancing age. 

The current study presents findings on the average modified Baumann's angle, which was measured to be 18.25±4.25° in males and 17.06±4.20° in females. In the study conducted by Keenan and Clegg [12], the average Baumann's angle was found to be 73.6±8.73° for males and 75.6±5.78° for girls. In the study conducted by Awasthi et al. [8], the average Baumann's angle was found to be 76.0±4.44° for males and 74.0±5.37° for females. During the examination conducted by Madison et al. [10], it was observed that the average Baumann's angle value for male patients was 72.2°, whereas for female patients it was 72.6°. In the study conducted by Dai et al. [11], it was observed that the average Baumann's angle range in males was 72.4±4.6°, while in females, the average Baumann's angle was 72.9±5.9°. In the study conducted by Leung KK [13], the average Baumann's angle was found to be 70.1° for males and 69.9° for females.

In our investigation, there was no statistically significant disparity observed in the mean modified Baumann's angle between male and female participants. In a prior investigation conducted by Keenan and Clegg [12], it was shown that there was no statistically significant disparity in the average Baumann's angle when comparing boys and girls across different age groups. This observation aligns with the findings of Dai et al. [11], who did a comparable study with individuals aged 2-12 years. In the study conducted by Awasthi et al. [8], no statistically significant difference was found in Baumann's angle between males and females. The studies of Madison et al. [10] and Williamson et al. [9] found no significant difference in Baumann's angle values across age groups or sex. This discrepancy was similarly comparable to the research conducted by Leung KK [13] in the Chinese community of Hong Kong.

The average modified Baumann's angle on the dominant and non-dominant sides was determined to be 17.07±4.41° and 17.05±4.06°, respectively, in females. Our study found that this difference was not statistically significant, which aligns with the findings of Keenan and Clegg [12], who also observed no significant difference in Baumann's angle between either side in their study. A relatively small negative and statistically significant association was observed between the modified Baumann's angle and the Carrying angle. This discovery exhibited similarity to the findings of Dai et al. [11], who conducted a study of a similar nature within the age range of 2-12 years. This observation coincides with the findings reported by Smajic et al. [14], who conducted a retrospective-prospective study involving children aged 14 years with supracondylar humerus fractures.

No statistically significant distinction was observed between the clinical and radiological carrying angles, which coincides with the results reported by Terra et al. [15]. Therefore, it may be inferred that a comprehensive clinical measurement is equally effective as a radiograph in giving adequate information for assessing the carrying angle of the elbow. A weakly negative and statistically significant connection was observed between modified Baumann's angle and both arm length and forearm length. We conducted a thorough search for recent scholarly literature pertaining to modified Baumann's angle. However, we were unable to locate any references that specifically explore the relationship between modified Baumann's angle and arm length or forearm length. Therefore, our study is likely the first to investigate and illustrate this link.

In this study, we observed a weak negative association between modified Baumann's angle and inter-epicondylar distance. However, it is important to note that this correlation did not attain statistical significance. We conducted a comprehensive search for recent academic literature about modified Baumann's angle but could not locate any references that specifically examine the relationship between modified Baumann's angle and inter-epicondylar distance. Therefore, our study is likely the first to investigate and illustrate this association, as per our knowledge.

## Conclusions

This study was an effort to evaluate the factors affecting modified Baumann’s angle in the paediatric age group, and the findings might be helpful in the management of elbow disorders and its reconstruction following trauma. A significant difference was found in the modified Baumann’s angle between dominant and non-dominant sides. The modified Baumann’s angle also showed a negative significant correlation with arm length and forearm length in our study. Those having secondary sexual characteristics had significantly lower mean values of modified Baumann’s angle as compared to those not having secondary sexual characteristics. The equations derived in this study will be helpful in the easy estimation of the modified Baumann’s angle and its difference between the two sides from simple measurements of the upper limb parameters.
